# Supplementary material for: Deletion of wfs1 Impairs Oligodendrocyte Precursor Cells Dorsal Distribution and Myelination Through the wfs1-hmgcs1 Axis in Zebrafish
Source: Biology (Basel). 2026 Aug 16;15(16):1402. doi: 10.3390/biology15161402 (PMC13510151; doi:10.3390/biology15161402)
Supplement: Supplementary file 1 [file biology-15-01402-s001.zip › File S1-Transcriptome sequencing data and differentially expressed genes (DEGs) in wfs1-- zebrafish at 5 dpf/2.KEGG/wfs1KOvsWT/DOWN/src/dre04141.html]

dre04141


- dre:564295

- Down regulated genes

564295\_2(-1.27307321576804)

- dre:100005358

- Down regulated genes

100005358(-3.40764150774661)

- dre:327197

- Down regulated genes

327197(-3.76691791748918)

- dre:565338

- Down regulated genes

565338\_1(-2.75872437189811)
- dre:559564

- Down regulated genes

559564(-1.18025564761092)

- dre:30671

- Down regulated genes

560210(-2.35591332225681)

- dre:393877

- Down regulated genes

393877(-1.08991261871184)

- dre:30671

- Down regulated genes

560210(-2.35591332225681)

- dre:323883

- Down regulated genes

323883(-4.8491500403135)

- dre:393877

- Down regulated genes

393877(-1.08991261871184)

- dre:386590

- Down regulated genes

386590(-2.05531811889352)

- dre:266757

- Down regulated genes

266757\_1(-1.86824458237205)

Close
